# Projection of E-Learning in Higher Education: A Study of Its Scientific Production in Web of Science

**DOI:** 10.3390/ejihpe11010003

**Published:** 2021-01-10

**Authors:** Jesús López-Belmonte, Adrián Segura-Robles, Antonio-José Moreno-Guerrero, María-Elena Parra-González

**Affiliations:** 1 Department of Didactics and School Organization, University of Granada, 51001 Ceuta, Spain; jesuslopez@ugr.es (J.L.-B.); ajmoreno@ugr.es (A.-J.M.-G.); 2 Department of Research Methods and Diagnosis in Education, University of Granada, 51001 Ceuta, Spain; elenaparra@ugr.es

**Keywords:** e-learning, higher education, bibliometrics, scientific production, Web of Science

## Abstract

E-learning is conceived as a purely virtual training approach. Different learning styles have been proliferated in recent years, especially now, due to the impact of COVID-19 in the educational field. The aim of this study is to discover the evolution of e-learning in higher education (ELHI) in scientific literature indexed on the Web of Science. Co-word analysis and bibliometric analysis was performed. A total matrix of 1261 documents was analyzed through SciMAT software. The results revealed that studies on ELHI are written in English and presented by conference papers. The main source of publication for the conferences is EDULEARN proceedings, while the journal source is *Procedia*-*Social and Behavioral Sciences*. Spain is the country with the highest volume of production. It is concluded that research on ELHI use does not have an established line of research, due to its recent creation and the lack of related research. The bibliometric analysis specifies that the research is oriented towards knowing the level of acceptance and application of the pedagogical method in the teaching and learning processes.

## 1. Introduction

Information and communication technologies (hereinafter ICT) are deeply rooted, widely accepted, and developed in today’s society [1]. The field of education, in particular, has not been left out of this technological projection, so it was nurtured, and it benefited from the continuous advances produced [2]. The inclusion and development of educational technology has allowed the emergence and evolution of various resources that serve to enhance and improve teaching and learning processes [3].

In this sense, new technological means have made it possible to generate new learning environments and methodologies [4,5]. In addition, they have promoted the development of various digital and innovative resources that complement and stimulate the training action carried out among educational agents, assuming new roles and functions of both the teacher and the students [6,7].

As a consequence of technological development in the field of education, the digital competence of teachers and students is presented as a relevant factor for the development of good practices with ICTs [8,9,10]. This competence is of great relevance for the effective realization of an instructional process in a digital plane, far from the classroom, as is the case of e-learning [11]. On the other hand, recent studies verify that current teachers do not have the skills and abilities necessary to carry out teaching and learning processes in digital environments [12,13,14], which makes it difficult to carry out digital teaching [15].

E-learning can be found within the broad spectrum of innovative learning methodologies. This training approach is conceived as a virtual instruction, carried out through content management platforms to carry out the teaching and learning process in a digital environment [16]. This training model emerged in 1996, creating consortia between universities to offer a digital educational alternative and, since then, this type of learning has proliferated in recent years [17], especially today, as a result of the impact of COVID-19 on the educational field [18]. E-learning, understood as a distance teaching method, allows the possibility of deploying a training plan of both a synchronous and asynchronous nature [19].

There are several advantages that e-learning has over other, more traditional teaching methods [20]. E-learning is understood as an instructional process focused on the student. In this case, the teacher’s role focuses on guiding student learning in digital environments [21]. In addition, the availability of the teaching materials and resources offered on the learning platforms allow students to deploy their training anywhere, and at any time, even cooperatively [22]. It is considered a type of adaptive learning, since it adapts to the particularities and learning rhythms of students. In this sense, this mode of instruction gives flexibility to the teaching and learning process [23].

This type of teaching allows greater communicative interaction between educational agents, by being able to emit messages through the platforms established by educational centers [24]. In addition to the advantages it presents for students, for the teaching community it also reports a series of potentialities. Teachers have specified in a digital space all training and resources [25]. They have all the tasks and information of the students [26]. Likewise, teachers can establish fluid channels of communication through private messages, discussion forums and even videoconferences [27].

Despite the potentialities reflected, e-learning also has limitations or disadvantages [28]. This innovative approach reduces physical contact between people [29]. It needs a set of technological requirements for its effective development (computer, mobile devices, internet connection, among the most prominent) [30]. In addition, this training modality requires certain digital skills [31]. Moreover, e-learning requires that all of the teacher’s teaching materials be converted to digital format in order to be able to use them through the established platform [32]. Likewise, it requires great autonomy and responsibility from the students to follow a distance training process [33].

After analyzing the literature on e-learning, it was observed that there are no studies that have analyzed the construct in a comprehensive manner. This is, from a bibliometric perspective, based on the scientific mapping of published studies. This problem, which has arisen in the impact literature, is intended to minimize with this work, presenting the importance and evolution of the concept of e-learning, specifically in higher education spaces. This research also offers the themes that e-learning runs through, as well as the way forward in coming years, revealing trends that are on the rise.

## 2. Materials and Methods

Due to the importance of the subject today, the purpose of this work is to know the evolution of e-learning in the higher education stage in the scientific documents registered in the Web of Science (WoS) database.

In this research, the concepts “e-learning” and “higher education” (ELHI) in WoS are studied. The analysis of this work was developed through scientific mapping [34,35,36,37].

The purpose of this research is to reveal the evolution of ELHI through the analysis of WoS export documents. In addition, it focuses on knowing how scientific production performs and evolves, as well as specifying the most influential topics and authors.

The research methodology used is bibliometrics. Bibliometrics allows to analyze different bibliographic characteristics in documents and the analysis of diverse features of scientific activity. This kind of analysis is important to scientific publications as a tool that allows qualifying the quality of the knowledge-generating process and the impact of this process on the scientific background [38]. Specifically, a co-word analysis has been developed [39]. In the same way, various indicators and indices frequently used in this type of study have been analyzed, such as the h, g, hg, and q^2^ index [40]. This is to design maps that contain nodes that represent the subdomains of the concepts linked to ELHI. All of this will allow the study of its thematic evolution.

The research procedure has been carried out in several stages: (1) selection of the database to be analyzed (WoS), considered the most important scientific database; (2) choice of keywords under study (“e-learning” and “higher education”); (3) construction of the search equation (“e-learning” (TITLE) AND “higher education” (SUBJECT)). Several inclusion criteria were also applied: year of publication (all from its origin); language (*n* ≥ 13); knowledge area (*n* ≥ 89); type of document (*n* ≥ 70); institutions (*n* ≥ 12); authors (*n* ≥ 5); source of origin (*n* ≥ 34); countries (*n* ≥ 74); citation (the four most cited references ≥ 100). These actions reported 1305 publications. The cut-off values for the criteria are established with those results whose nodes are formed by more than one document or value.

Publications indexed in 2020 and repeated or poorly registered documents were established as exclusion criteria. Moreover, 1261 publications conformed final analysis matrix. This figure was classified into three-time intervals (I1 = 2000–2011; I2 = 2012–2015; I3 = 2016–2019) that have been prepared under the criterion of documentary similarity.

All presented analysis was carried out with the citation reports tool of WOS and SciMAT software.

## 3. Results

### 3.1. Diachronic Analysis and Scientific Production

**Figure 1:**
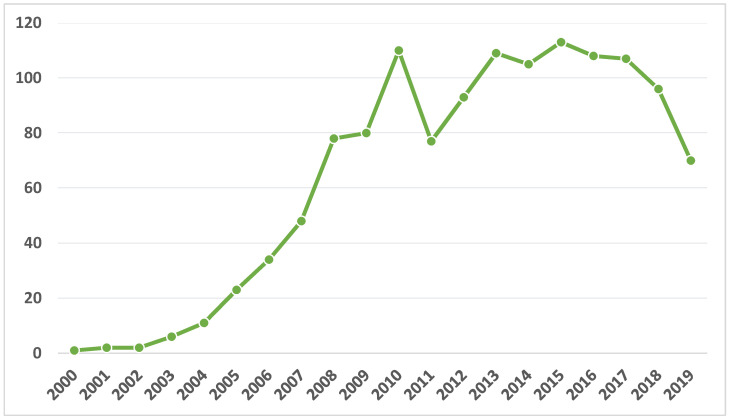
Diachronic evolution of e-learning in higher education (ELHI).

The set of documents related to ELHI in Web of Science is 1261. The start of production dates back to the year 2000. From that date until 2010, the growth in the volume of documents was high (being increasing and constant throughout the period). In 2011 the scientific production decreased compared to the previous year, but in 2012 and 2013, it increased again. From that same year until 2017, the production was constant (concerning quantity). However, in 2018 and 2019, production fell to levels close to 2008. Peak production occurred in 2015 (Figure 1).

The language, mainly used by researchers, is English. All other languages are far removed from it (Table 1).

The studies on ELHI are collected mainly in the knowledge area Education Educational Research, being far from the other areas (Table 2).

Both papers and research articles are the main types of documents used by researchers to show their findings. The number of papers stand out, confirming the fact that this field of study is of a recent appearance in the scientific community (Table 3).

The Universitat Oberta de Catalunya is the leading institution in ELHI-related studies. The fact that the most prolific universities are Spanish stands out (Table 4).

The author with the highest level of scientific production on ELHI is Pelet, J.E. The rest of the authors are at a close distance, which shows the variety and number of authors who study the field under analysis (Table 5).

The main sources collecting research on ELHI are two books of conference proceedings, with EDULEARN as the main reference. This fact coincides with the data offered previously on the source of production. In this case, the main journal that publishes studies on this subject is Procedia Social and Behavioral Science (Table 6).

Two countries are the reference countries in this field of study in scientific production. Spain is in first place, closely followed by England (Table 7).

In ELHI’s field of study, the most widely cited work has 234 citations [41]. The second work has 205 citations [42] (Table 8).

### 3.2. Development of the Structure and Thematic Axis of ELHI

**Figure 2:**
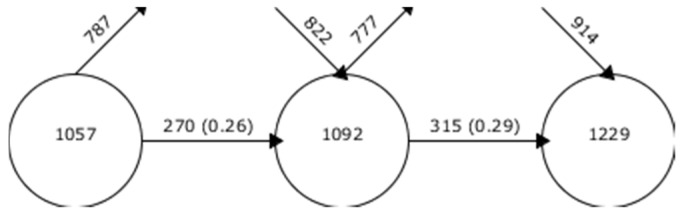
Keywords continuity between intervals.

The evolution of keywords shows the number of keywords collected in a given period. In addition, it also shows the keywords that go out and come in a certain period. In this study, the level of keyword matching between contiguous periods is low, and it increased from its beginnings until 2019. This shows that the line of investigation is not established, although it is beginning to be established (Figure 2).

The academic performance presents the most valuable subjects in each of the established time periods. These values are based on bibliometric indicators of various kinds (h, g, hg, and q2). Academic performance is shown according to established time periods. In the first interval (2000–2011), the subject with the highest bibliometric value is “education”. In the second interval (2012–2015), “e-learning” is the most important. In the third interval (2016–2019), it is “extension” and “students” (Table 9).

**Figure 3:**
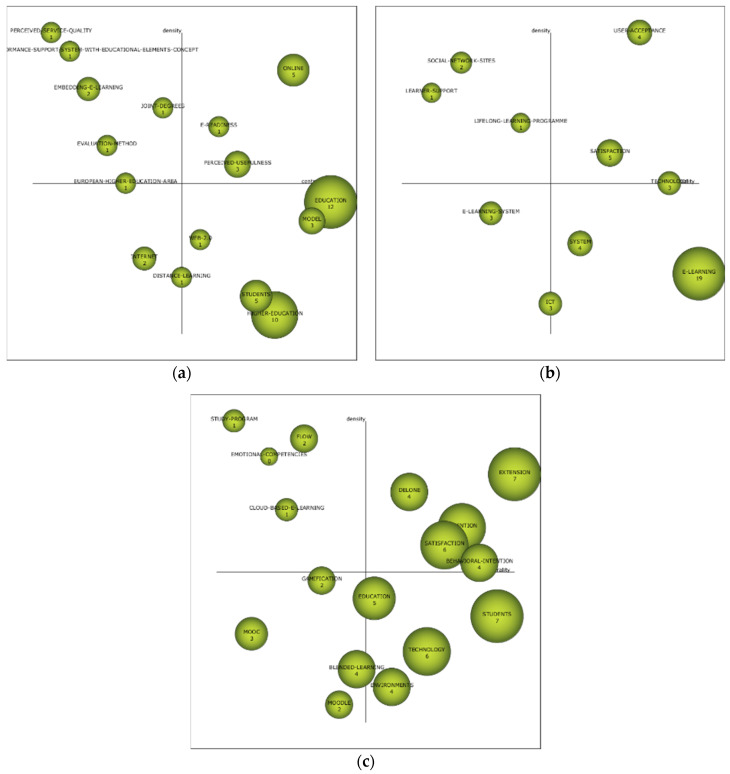
ELHI Strategic diagram base on index-h. Note: (**a**) period 2000–2011; (**b**) period 2012–2015; (**c**) period 2016–2019.

Thematic diagrams provide data on the relevance of the different topics in a given time period. The coordinate axis shows the level of centrality and density. The former analyzes the relational strength of external links. The second analyzes the relational strength of internal links. The study of the three diagrams shows that no single theme is repeated as the driving force in the three periods. Only “satisfaction” stands out, which appears in the second and third periods (Figure 3).

In the first interval (2000–2011), the driving themes are “perceived-usefulness”, which is related to “impact”, “technology-acceptance-model”, and “user-acceptance”; “e-readiness”, which is based on “measuring-e-readiness”, “cultural-aspects”, and “IT”; and “on-line” which is oriented towards “qualitative-differences”, “knowledge-work”, “teaching-quality”, “technology”, “satisfaction”, “conceptions”, “teaching/learning-strategies”, and “face-to-face”. In this period, although ELHI is analyzed, it seems that the research interest is more oriented towards distance learning than e-learning.

In the second interval (2012–2015), the driving themes are “used-acceptance”, which is related to “perceived-ease”, “extension”, “national-culture”, “information-technology”, “model”, “behavioral-intention”, “self-efficacy” and “technology-acceptance”; and “satisfaction”, which focuses on “information-systems-success”, “continuance-intention”, “instructor”, “critical-success-factors”, “web”, “management”, and “web-based-e-learning-system”. In this period, the evaluation and acceptance of the e-learning method, mainly oriented to technological resources, takes on an important nuance.

In the third and final interval (2016–2019), the driving themes are “DeLone” which is related to “McLean-model”, “information-systems-success”, “attitudes”, “e-learning-system”, and “management-systems”; “extension”, which is oriented towards “unified-theory”, “determinants”, “students-behavioral-intention”, “information-technology”, “technology-acceptance-model”, “user-acceptance”, “usage” and “variables”; “intention” which is based on “acceptance”, “information-systems-continuance”, “trust”, “computer”, “model”, “adoption”, “self-efficacy” and “perceived-ease”; “satisfaction”, which relates to “e-learning-success”, “students-perspective”, “public-administration-education”, “quality-of-e-learning-system”, “critical-success-factors”, “performance”, “context” and “use”; and “behavioral-intention” which focuses on “technology-acceptance”, “TAM”, “system-characteristics”, “perspective”, “preservice-teachers”, “structural-equation-modeling”, “university-students”, and “perceived-usefulness”. In this period, the e-learning method is more relevant, the behavior of the subjects when faced with the pedagogical method and the relationship between the people involved in the pedagogical act. Furthermore, in this period, the themes “MOOC”, “gamification”, “blended-learning”, and “Moodle” must be taken into account, given that their location in the diagram places them as unknown themes. In other words, the topics may become the next driving force, or they may disappear from the scientists’ lines of research.

### 3.3. Scientific Evolution of ELHI

The scientific evolution provides information on the relationship between the themes of the recognized contiguous periods. To establish these relationships, the Jaccard index has been taken into account. The relationships can be of two types: continuous line and discontinuous line. The thickness of the lines shows the strength of the relationship between the themes.

**Figure 4:**
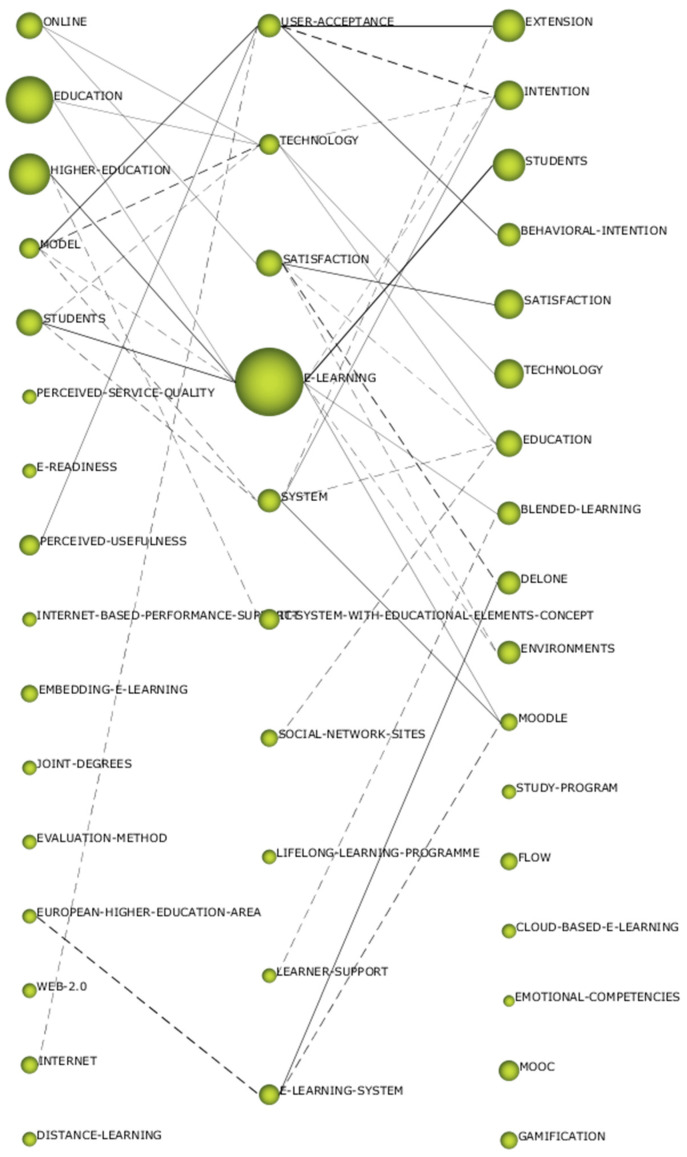
Scientific evolution of ELHI by h-index.

The scientific evolution of ELHI presents a conceptual gap. This is due to the fact that there is not one theme that is repeated in the three periods. Even so, two lines of research can be glimpsed, on the one hand that established by “model-user/acceptance-extension/behavioral-intention” and—on the other—that of “students-e/learning-students”. In other words, studies in this field of study are mainly oriented towards the acceptance of the pedagogical model and in the students themselves. Furthermore, as can be seen in Figure 4, there is a shift from elements of distance learning to aspects of e-learning. It can also be seen how terms such as social networks, learning for life, and learning platforms appear, which shows the variety of resources and the purpose of this type of teaching method. It should be borne in mind that there are more conceptual than non-conceptual connections.

### 3.4. Most Relevant Authors

**Figure 5:**
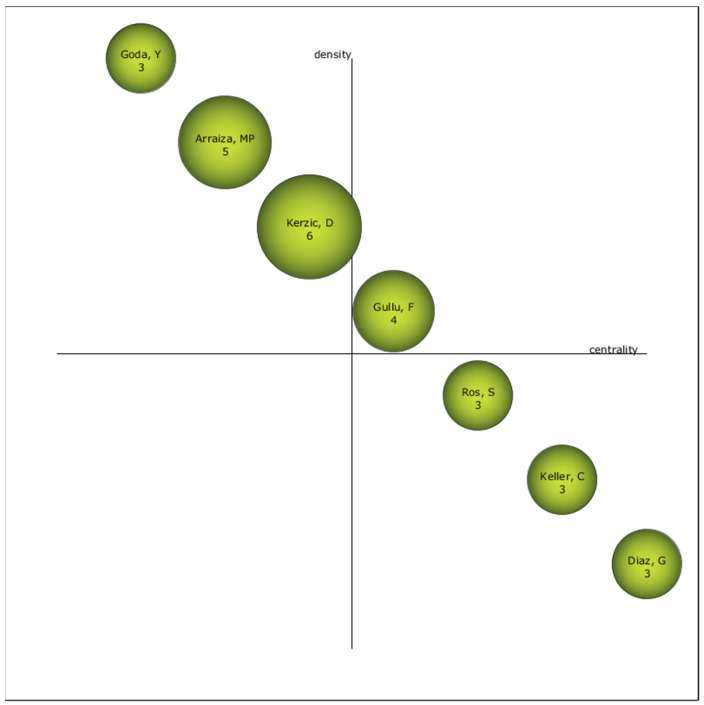
Most relevant scientists by h-index.

The author considered as the driving force in the ELHI study is Gullu, F. (Figure 5), because of his position in the diagram.

## 4. Discussion

The ELHI field of study is relatively young, as it began in 2000 according to WoS. From that date to 2019, the level of production was irregular, with a great deal of incidence by the scientific community from 2000 to 2010, and then remaining constant over time. Since 2018, there was a downward trend in production, although with the health crisis caused by the COVID-19, it will probably rise again, given that it is currently the model used to develop all types of education, both university and non-university levels.

The studies on ELHI are written in English and presented in conference papers. The area of knowledge where research is based on is Education Educational Research. The most important institution in this field of study is the Universitat Oberta de Catalunya (Open University of Catalonia), with Spanish institutions being the most productive. Pelet, J.E. is the author with the largest number of productions, although Gullu, F. stands out as the most relevant in this field of study. The main source of publication for the conferences is EDULEARN proceedings, while the journal source is Procedia Social and Behavioral Sciences. Spain has the largest scientific production on ELHI. The most cited manuscript is [41], with 234 citations.

If we look at the co-word analysis, we can see that this field of study does not have a solid line of investigation recognized, given that the level of coincidence of key words between the established periods is low. Even so, it can be seen that a line of research is beginning to be established in this field of study, given that, over time, the number of key words coinciding between periods is increasing.

Academic performance reiterates and confirms this fact, given that there is not one theme that repeats in all three periods, not even in two. This analysis shows that the topics dealt with in this field of research are oriented towards education, online teaching, and students.

With regard to the most relevant topics in this field of study, it is shown that there is an evolution over time, with varying interest and focus on the part of the researchers. In the first period, interest is focused more on distance learning than on online learning itself, given that it uses terms that are not typical of the e-learning method. In the second period, this fact changes radically, given that interest is focused on the assessment and acceptance of e-learning by those involved in this teaching process. In the third period, the line of the second period is maintained a little, although the researchers extend their field of interest to the intention of those involved in the training process.

Over the next few years, the themes of “MOOC”, “gamification”, “blended-learning” and “Moodle” should be kept in mind when researching the use of e-learning in higher education, and could be the next driving force.

The thematic evolution once again confirms what was indicated previously—that is to say, there is no solid and stable line of research in this field. Moreover, there is a conceptual research gap related to ELHI. Even so, there are two lines that have focused more interest on the part of the scientific community. These timelines are “model-user/acceptance-extension/behavioral-intention” and “students-e/learning-students”. In other words, studies in this field of study are mainly oriented towards the acceptance of the pedagogical model and in the students themselves.

## 5. Conclusions

It can be concluded that research on the use of ELHI does not have an established line of investigation. This bibliometric study indicates that the subject is quite new in the Web of Science database. That is to say, studies on this line of research have begun to be registered recently. The bibliometric analysis specifies that the research is oriented towards knowing the level of acceptance and application of the pedagogical method in the teaching and learning processes. This information offered here can guide researchers and professors in their work. The prospective of this research consists of offering the scientific community new trends in this field of study. In addition, the aim is to show the educational community the latest trends on this method of teaching and learning.

As for the limitations of this research, we must say that there are several. Firstly, generating the database in WoS requires a great deal of effort for researchers. This is because the entire scientific production must be read to determine whether it follows the inclusion criteria set out in the PRISMA (Preferred Reporting Items for Systematic Reviews and Meta-Analyses) protocol. Secondly, the time distribution has been carried out, taking into account the criterion of equity. An attempt has been made to collect an even number of manuscripts in each of the established time periods. Thirdly and finally, the criteria for inclusion have been established at the discretion of the researchers, which have sought to present results that are in line with each other in terms of size and relevance. In this case, the data shown in this study should be considered with caution. The change in the inclusion criteria may slightly change the data shown in this research. Future lines of research include analyzing the e-learning method in other educational stages, such as compulsory secondary education, baccalaureate, or vocational training.

## Figures and Tables

**Table 1:** Scientific languages used.

Language	n
English	1200
Spanish	42
Portuguese	14

**Table 2:** Knowledge area.

Knowledge Area	n
Education Educational Research	832
Computer Science Information Systems	172
Computer Science Interdisciplinary Applications	171
Computer Science Theory Methods	105
Engineering Electrical Electronic	90

**Table 3:** Types of documents.

Type of Document	n
Proceedings	659
Article	569
Book chapters	71

**Table 4:** Institutions.

Institutions	n
UOC Universitat Oberta de Catalunya	22
University of Seville	16
Polytechnic University of Madrid	13

**Table 5:** Most prolific authors.

Authors	n
Pelet, J.E.	8
Aristovnik, A.	6
Dobre, I.	6
Dukic, D.	6
Kerzic, D.	6
Tomazevic, N.	6
Umek, L.	6

**Table 6:** Sources of origin.

Sources of Origin	n
EDULEARN Proceedings	64
INTED Proceedings	40
Procedia Social and Behavioral Sciences	36
E-learning and Software for Education	35

**Table 7:** Countries.

Countries	n
Spain	147
England	141
Romania	82
United States	75

**Table 8:** Most Cited Articles.

Reference	Citation
[41]	234
[42]	205
[43]	133
[44]	114

**Table 9:** Thematic performance in ELHI.

**Time Interval 2000–2011**						
**Denomination**	**Works**	**Index h**	**Index g**	**Index hg**	**Index q2**	**Citations**
Online	9	5	5	5	16.43	241
Education	32	12	20	15.49	16.25	412
Higher-education	30	10	18	13.42	13.42	361
Model	8	3	6	4.24	16.52	371
Students	10	5	7	5.92	10	151
Perceived-service-quality	2	1	1	1	2.65	7
E-readiness	4	1	1	1	1	2
Perceived-usefulness	3	3	3	3	10.25	103
Internet-based-performance-suport-system-with-educational-elements-concept	2	1	1	1	1	1
Embedding-e-learning	2	2	2	2	6	20
Joint-degrees	2	1	1	1	1	1
Evaluation-method	2	1	2	1.41	2	5
European-higher-education-area	3	1	1	1	1	1
Web-2.0	4	1	1	1	1	1
Internet	3	2	3	2.45	13.49	178
Distance-learning	4	1	1	1	1	2
**Time Interval 2012–2015**						
**Denomination**	**Works**	**Index h**	**Index g**	**Index hg**	**Index q2**	**Citations**
User-acceptance	9	4	7	5.29	6.63	55
Technology	19	3	9	5.2	9.49	89
Satisfaction	11	5	8	6.32	21.68	463
E-learning	134	19	32	24.66	27.91	1199
System	7	4	5	4.47	6	44
ICT	7	3	5	3.87	7.35	50
Social-network-sites	2	2	2	2	2	4
Lifelong-learning-program	4	1	1	1	2.65	7
Learner-support	2	1	1	1	1.41	2
E-learning-system	4	3	4	3.46	5.48	32
**Time Interval 2016–2019**						
**Denomination**	**Works**	**Index h**	**Index g**	**Index hg**	**Index q2**	**Citations**
Extension	25	7	12	9.17	9.9	161
Intention	19	6	11	8.12	11.49	133
Students	121	7	10	8.37	8.37	258
Behavioral-intention	13	4	6	4.9	7.48	51
Satisfaction	14	6	12	8.49	13.86	147
Technology	14	6	8	6.93	7.35	78
Education	10	5	7	5.92	5	65
Blended-learning	10	4	6	4.9	4.47	44
DeLone	7	4	6	4.9	11.31	92
Environments	9	4	7	5.29	8.25	67
Moodle	8	2	4	2.83	4.47	17
Study-program	3	1	1	1	1.41	2
Flow	2	2	2	2	8.49	46
Cloud-based-e-learning	4	1	2	1.41	2.24	6
Emotional-competencies	2	0	0	0	0	0
MOOC	4	3	3	3	3.87	15
Gamification	3	2	2	2	3.74	9

## Data Availability

Data is contained within the article.
